# Complete m6A and m4C methylomes for group B streptococcal clinical isolates CJB111, A909, COH1, and NEM316

**DOI:** 10.1128/mra.00733-23

**Published:** 2023-12-15

**Authors:** Haider S. Manzer, Kelly S. Doran

**Affiliations:** 1 Department of Immunology and Microbiology, University of Colorado Anschutz Medical Campus, Aurora, Colorado, USA; Indiana University, Bloomington, Bloomington, Indiana, USA

**Keywords:** GBS, methylation, m6A, m4C, 6mA, 4mC, epigenetics

## Abstract

Group B *Streptococcus* (GBS) is known to colonize the female reproductive tract and causes adverse pregnancy outcomes and neonatal disease. DNA methylation is a common mechanism for both phage defense and transcriptional regulation. Here, we report the m6A and m4C methylomes of four clinical GBS isolates, CJB111, A909, COH1, and NEM316.

## ANNOUNCEMENT

*Streptococcus agalactiae* (group B *Streptococcus*; GBS) asymptomatically colonizes the female reproductive tract in one-third of healthy adults (1, 2). GBS colonization can lead to numerous adverse pregnancy outcomes and is a leading cause of neonatal sepsis, pneumonia, and meningitis worldwide (3). The roles of bacterial DNA methylation in phage defense and competence as part of restriction-modification systems are well defined (4 – 11). The ability for DNA methylation, especially in promoter regions, to transcriptionally alter gene expression has also been well studied in recent years (12 – 29). While 5mC DNA methylation is most common in eukaryotes, m6A and m4C represent the bulk of prokaryotic DNA methylation (12, 19, 21, 30 – 32). Thus, we used PacBio single-molecule real-time (SMRT) sequencing for whole-genome identification of m6A and m4C methylation at a single-nucleotide resolution in four well-researched GBS clinical isolates: CJB111, A909, COH1, and NEM316. CJB111 is a serotype V strain isolated by Dr. Carol J. Baker from the blood of an infant with late-onset sepsis in Houston, TX, USA, 1990 (33). A909 is a serotype Ia strain isolated from the blood of a septic neonate in 1934 and is part of Rebecca Lancefield’s strain collection (34). COH1 and NEM316 are both serotype III strains isolated from the blood of a neonate and from a fatal case of neonatal septicemia, respectively (35, 36). We obtained these strains from Dr. Carol Baker (CJB111), Dr. Glen Tamura (COH1), Dr. Victor Nizet (A909), and ATCC (NEM316) and sequenced our master stocks which are only one to two subcultures removed from the strains we received.

All four strains were grown overnight in Todd-Hewitt broth standing at 37°C. DNA was isolated using the Qiagen Gentra Puregene Yeast/Bact. Kit. DNA concentration and quality were confirmed using a NanoDrop spectrophotometer and Qubit fluorometer and the Qubit dsDNA BR Assay Kit. DNA was sheared on a Diagenode Megaruptor and size selected using a Sage BluePippin before library preparation using the SMRTbell prep kit 3.0 following PacBio guidelines. Then, 20 hours of HiFi sequencing with kinetic data were performed using a SMRT Cell 8M, PacBio Sequel II sequencer, and SMRTLink v.11 at the DNA Sequencing Center of Brigham Young University, along with adapter and primer trimming and demultiplexing with Lima v.2.71. Error-corrected reads below a Phred scaled quality value of 20 were filtered out, and remaining reads were mapped to reference genomes with pbmm2 v.1.12.0 for each strain (CJB111: CP063198; A909: 007432; COH1: HG939456; NEM316: 004368) with methylation base calling using the microbial genome analysis application included in SMRTLink v.12.

**Fig 1:**
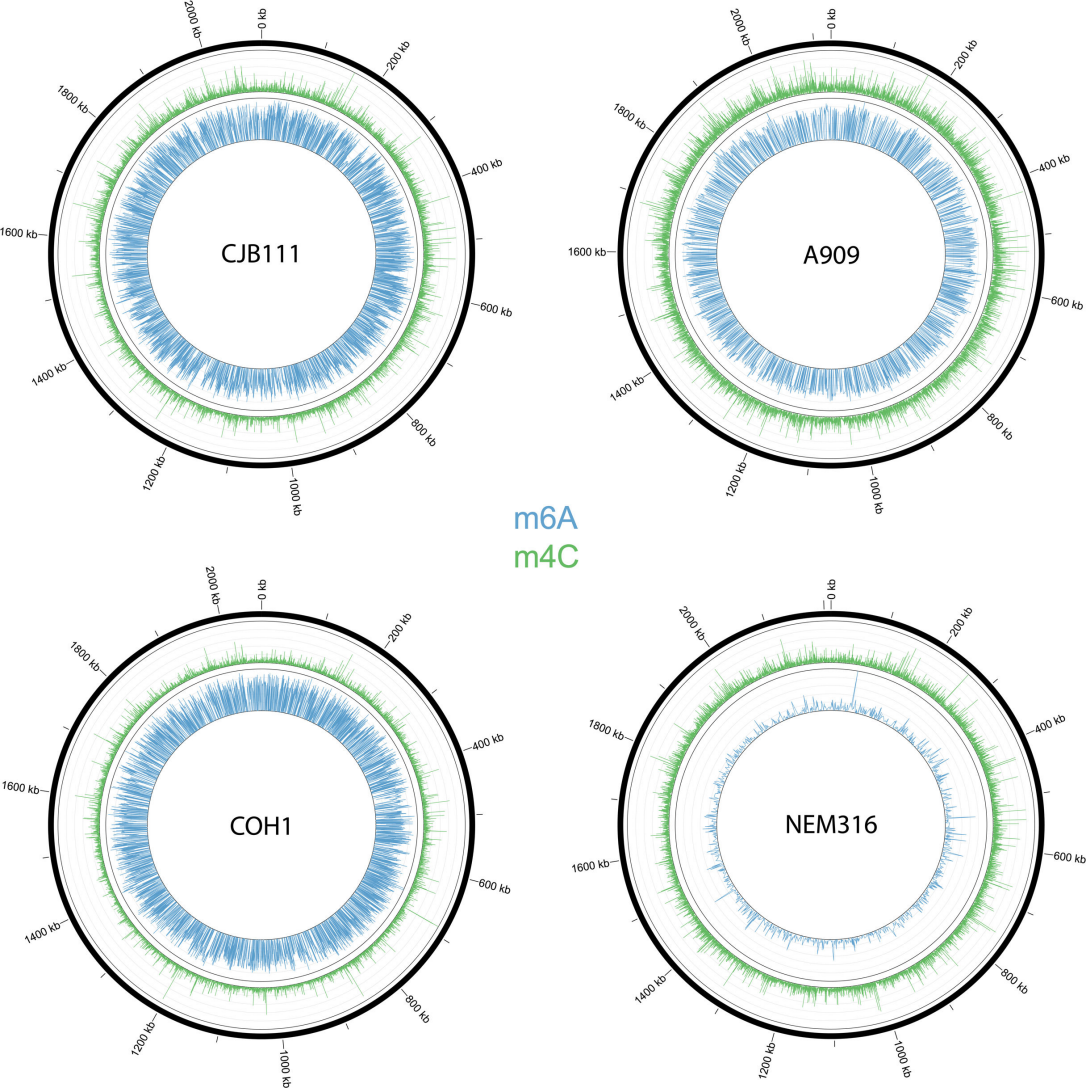
Circos plots of DNA methylation. Blue represents m6A, and green represents m4C. Height of each peak represents prediction strength using Phred-transformed scores per methylation call provided by the SMRTLink v.12 microbial genome analysis application. The four GBS strains are indicated. Circos plots were generated with Circos v.0.69-9.

Sequencing statistics and motif identification are provided in Table 1. The per-base methylation for all four strains is summarized in Fig. 1. Rates of m4C DNA methylation are consistent between strains, while rates of m6A DNA methylation appear more variable, with NEM316 entirely lacking a consistently m6A-methylated motif. There is more m4C than m6A methylation in these strains; however, m6A motifs are generally more thoroughly methylated (>99% methylation rate).

**TABLE 1:** Methylation motifs and sequencing statistics^*a*^

	Methylation motifs								Sequencing statistics							
Strain	Motif	Type	# Detected	# In genome	% Methylated	Mean score	Mean interpulse duration ratio	Mean coverage	Genome length	GC%	m4C base calls (% of genome)	m6A base calls (% of genome)	# of reads	Mean read length (StDev)	Mean coverage (StDev)	% of reads <*Q*40
CJB111	GCS **A** G	m6A	2,413	2,419	0.998	1187.41	4.90	579.55	2,093,987	35.5	17,523 (0.84%)	5,412 (0.26%)	500,595	5171.4 (1855.8)	1212.8 (286)	97.8
	GCAA **A** T	m6A	1,609	1,610	0.999	871.29	4.14	560.01								
	BVN **C** YGVNCAD	m4C	454	821	0.553	228.76	2.15	575.87								
A909	GCAA **A** T	m6A	1,637	1,637	1.000	582.72	4.48	209.85	2,127,839	35.6	15,114 (0.71%)	2,869 (0.14%)	194,649	4674.6 (1784.2)	419.5 (60.9)	97.9
	GN **C** YGVNYRD	m4C	415	1,214	0.342	162.39	2.24	213.72								
	BMN **C** YGVNCAD	m4C	210	629	0.334	154.81	2.14	213.20								
	GR **C** YCANCRD	m4C	78	182	0.429	143.05	2.05	212.17								
	GN **C** TGNTCTANNNW	m4C	14	18	0.778	133.21	2.01	216.43								
	ANNNNNNGGGA **C** CGA	m4C	13	14	0.929	118.54	1.91	210.00								
COH1	TGG **A** G	m6A	3,255	3,259	0.999	1208.92	4.95	584.09	2,065,074	35.4	17,272 (0.84%)	4,797 (0.23%)	500,799	5122.8 (1965.6)	1222.5 (206.2)	97.8
	BVN **C** YGVNCAD	m4C	411	778	0.528	240.17	2.18	592.85								
	GV **C** YSRNYD	m4C	668	2,549	0.262	209.64	2.06	591.55								
	MNNNNNHNNNNKGG **C** CCH	m4C	58	90	0.644	246.33	1.93	603.45								
	DNNNBNN **C** TCAGCA	m4C	60	112	0.536	177.77	1.95	586.55								
	HNDGGG **C** CTNNNNNH	m4C	40	84	0.476	165.85	1.92	601.50								
NEM316	GN **C** YGVNYRD	m4C	579	1,289	0.449	206.26	2.16	426.10	2,211,485	35.6	17,312 (0.78%)	1,508 (0.07%)	370,441	5132.6 (2000.6)	841.8 (106.2)	97.8
	BMN **C** YGVNCAD	m4C	276	642	0.430	178.43	2.02	426.12								
	GV **C** TCANCRD	m4C	96	202	0.475	159.41	1.95	426.66								

^*a*^ Methylated nucleotide in each motif is indicated by bolding and underlining.

## Data Availability

The raw sequence reads are accessible under Sequence Read Archive accession no. CJB111: SRX20539489, A909: SRX20539490, COH1: SRX20539491, and NEM316: SRX20539492; BioProject accession no. PRJNA977207; and BioSample accession no. CJB111: SAMN35521425, A909: SAMN35521426, COH1: SAMN35521427, and NEM316: SAMN35521428.
